# Effect of Gait Speed on Trajectory Prediction Using Deep Learning Models for Exoskeleton Applications

**DOI:** 10.3390/s23125687

**Published:** 2023-06-18

**Authors:** Rania Kolaghassi, Gianluca Marcelli, Konstantinos Sirlantzis

**Affiliations:** 1 School of Engineering, University of Kent, Canterbury CT2 7NT, UK; g.marcelli@kent.ac.uk; 2 School of Engineering, Technology and Design, Canterbury Christ Church University, Canterbury CT1 1QU, UK; konstantinos.sirlantzis@canterbury.ac.uk

**Keywords:** artificial intelligence, gait speeds, deep learning, exoskeletons, forecasting, gait, prediction, extrapolation, kinematics

## Abstract

Gait speed is an important biomechanical determinant of gait patterns, with joint kinematics being influenced by it. This study aims to explore the effectiveness of fully connected neural networks (FCNNs), with a potential application for exoskeleton control, in predicting gait trajectories at varying speeds (specifically, hip, knee, and ankle angles in the sagittal plane for both limbs). This study is based on a dataset from 22 healthy adults walking at 28 different speeds ranging from 0.5 to 1.85 m/s. Four FCNNs (a generalised-speed model, a low-speed model, a high-speed model, and a low-high-speed model) are evaluated to assess their predictive performance on gait speeds included in the training speed range and on speeds that have been excluded from it. The evaluation involves short-term (one-step-ahead) predictions and long-term (200-time-step) recursive predictions. The results show that the performance of the low- and high-speed models, measured using the mean absolute error (MAE), decreased by approximately 43.7% to 90.7% when tested on the excluded speeds. Meanwhile, when tested on the excluded medium speeds, the performance of the low-high-speed model improved by 2.8% for short-term predictions and 9.8% for long-term predictions. These findings suggest that FCNNs are capable of interpolating to speeds within the maximum and minimum training speed ranges, even if not explicitly trained on those speeds. However, their predictive performance decreases for gaits at speeds beyond or below the maximum and minimum training speed ranges.

## 1. Introduction

Exoskeletons are robotic devices utilised to enhance the strength and ability of unimpaired users, restore movement, and assist in rehabilitating people with pathological gaits [1,2,3]. Exoskeletons have varying control strategies [4] and are broadly categorised into weight-bearing devices that transfer the load to the ground and joint-targeting devices that deliver targeted assistance to specific joints [1].

Some of these exoskeletons integrate Artificial Intelligence (AI) models into their control systems [5] to perform tasks such as gait phase classification [6], prediction of joint kinetics and kinematics [7,8], locomotion mode classification and intention prediction [9,10], environment detection [11], and reference gait personalisation [12].

AI models require training on datasets and in the case of exoskeleton applications, the training dataset could include gait parameters such as joint kinetics, joint kinematics, foot pressure, and muscle activity [5]. AI models utilise these parameters as input to make predictions or classifications, depending on the task. It is crucial for the training dataset to be a representative sample of the data that the exoskeleton is expected to receive as input during real-life operation. Depending on the data used for training and testing the model, we can develop individualised (dependent), generalised (independent), and semi-dependent models [13]. A dependent model needs to be trained on data from an exoskeleton user before using the exoskeleton. An independent model, on the other hand, does not require training on data from each exoskeleton user but is instead trained on data from multiple individuals. Semi-dependent models combine both approaches, where a model is trained on data from multiple individuals but fine-tuned using data from the specific user before using the exoskeleton [13]. Although individualised models have demonstrated higher accuracy in performance compared to generalised models [13], it is not always possible or practical to fine-tune models for each user, which is why generalised models are sometimes adopted.

It is essential to consider how the models, often trained on data from able-bodied users, would perform when controlling assistive and rehabilitative exoskeletons for users with pathological gaits. The target users of these exoskeletons typically walk at slower gait speeds [14]. For instance, able-bodied adults have walking gait speeds between 0.75 and 1.75 m/s, whereas adult stroke patients walk at much lower speeds, ranging between 0.08 and 1.05 m/s [15]. Additionally, over the course of a rehabilitation session, a patient’s speed may change, with their mean velocity increasing [16].

The main contribution of this study is to investigate the effect of speed on the prediction of gait trajectories for able-bodied users, as well as the performance of the models in predicting trajectories at gait speeds that are excluded from the training speed range (see Section 3 for details). We compare the performance of the models on gait speeds included in the training range, as well as speeds excluded from it. This investigation enables us to assess the generalisability of the models when tested on gaits from users walking at speeds that have not been included in the training dataset and to examine the influence of speed on gait. The findings of this study can serve as guidance for developers of exoskeletons, informing their decision on which speeds to include when collecting data for training AI models for exoskeleton control.

## 2. Literature

Gait speed is known to impact spatiotemporal parameters (cadence, step length, and stride length), joint kinetics, ground reaction forces, and joint kinematics [17]. The magnitude of this impact varies among children, young adults, and older adults [17]. The effect of gait speed on joint kinematics has been shown to be moderate to large [17]. In young adults, for example, gait speed has been shown to impact the minimum and maximum joint angle values, specifically increasing hip flexion, hip extension, knee flexion, and ankle plantar-flexion angles with higher speeds [17]. Furthermore, gait speed appears to have a more significant impact on the kinematics of children, whose gait patterns have not fully matured, compared to individuals from other age groups [17,18].

Since speed is a vital biomechanical determinant of gait patterns [14], it is essential to consider it when generating reference gait patterns for position-controlled exoskeletons. Fukuchi et al. utilised regression to generate normalised reference gait patterns that were speed-dependent. They aimed to establish a database of reference gait patterns at varying speeds that could be used to assess the gait patterns of individuals with gait pathologies [14] who often walk at lower speeds compared to able-bodied individuals [19,20]. Zaroug et al. assessed the performance of deep learning models in predicting lower limb kinematics at speeds 20% lower and higher than preferred walking speeds. The performance of their models decreased when predicting gait at slower speeds but increased when predicting gait at faster speeds [21]. Apart from speed, anthropometric parameters can also influence gait patterns [22,23,24]. Zou et al. [25] developed a two-step method for gait trajectory prediction based on an individual’s unique anthropometrics and desired speed during rehabilitation. Their approach consisted of a Gait Parameter Model (GPM), which is a neural network that selects gait parameters based on anthropometrics and speed, and a Gait Trajectory Model (GTM), which uses these parameters, along with kernelised movement primitives, to reconstruct the reference gait patterns for an exoskeleton. Han et al. [26] implemented Future Generative Adversarial Nets (F-SeqGAN) trained on varying gait speeds for gait trajectory prediction, even during acceleration, without the need to pre-define the input speed. Embry et al. [27] developed a basic model that can continuously predict joint kinematics based on gait phase, speed, and inclination.

Although many of the existing approaches test their models on the gait speeds included in the training speed range, only a few have explored how the models perform and extrapolate to speeds that are excluded from the training set. For the task of gait phase prediction, Lu et al. [28] observed a decrease in the performance of a long short-term memory (LSTM) network on speeds not included in the training set. However, their study was limited to only two subjects, and the training data were biased towards constant speeds. Meanwhile, Kang et al. [13] implemented neural networks for gait phase prediction and found that their models were capable of extrapolating to higher speeds, with the semi-dependent model outperforming the dependent and independent models. Their results were based on 10 subjects, but they only extrapolated to speeds 0.1 m/s higher than the training speed range. The two aforementioned studies assessed the ability of the models to classify gait phases on speeds excluded from the training speed range, but either had a low number of participants or extrapolated to speeds that were only slightly beyond the training speed range. Furthermore, no studies have investigated the task of gait trajectory prediction, where the performance of models is evaluated for gait speeds that are both lower and higher than the training speed range.

To address these gaps, our study aims to evaluate and compare the performance of deep learning models when tested on speeds that are both included and excluded from the training speed range. This assessment may help determine the transferability of the models to real-world applications, where an exoskeleton may need to operate in an environment with greater variability than what it was initially trained for.

## 3. Methodology

### 3.1. Overview

In our study, we evaluated how fully connected neural networks (FCNNs) for gait trajectory prediction extrapolate to speeds excluded from the training range. The FCNNs were trained to perform one-step-ahead predictions of joint kinematics, focusing on the angles of the hip, knee, and ankle for both the left and right legs. Predictions were based on a short input window of past joint kinematic values. We developed and trained four FCNNs using data obtained at varying gait speeds. Subsequently, we assessed the performance of the models by testing them on gait speeds included and excluded from the training speed range (refer to Table 1 for further details).

### 3.2. Data

**Figure 1:**
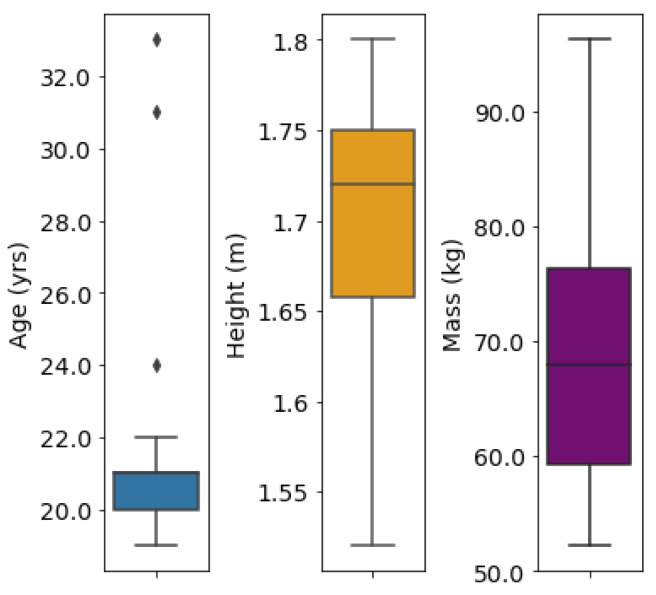
Box plots showing the demographics of the individuals in the training and testing sets, including age, height, and mass. The box represents the quartiles, the whiskers represent the interquartile range, and the points located outside the whiskers represent outliers in the dataset.

The FCNNs in our study were trained using an online gait dataset by Camargo et al. [29]. The dataset consists of gait data collected from 22 able-bodied individuals (age 21 ± 3.4 years, height 1.70 ± 0.07 m, mass 68.3 ± 10.83 kg). Gait patterns were recorded while walking on a treadmill at 28 different speeds (0.5 m/s to 1.85 m/s in 0.05 m/s increments). The dataset contains joint kinematic values including the hip, knee, and ankle angles of the left and right legs in the sagittal plane, which were used in our study. The joint angles were derived using OpenSim’s inverse kinematics tool based on motion capture data collected at a 200 Hz sampling frequency. For a visual representation of the demographic distribution of the dataset, refer to Figure 1. Additional details about the data processing procedures can be found in [29].

### 3.3. Pre-Processing

In order to evaluate the predictive performance of the FCNNs at speeds excluded from the training range, we segmented the data into three distinct speed ranges: low speeds ranging from 0.5 m/s to 1.0 m/s, medium speeds ranging from 1.05 m/s to 1.45 m/s, and high speeds ranging from 1.5 m/s to 1.85 m/s. These speed ranges were determined based on the low-, comfortable-, and high-speed ranges reported in the literature for young adults [17,30,31].

Four FCNNs were developed and evaluated in our study. They consisted of the same architecture (including the number of layers and nodes per layer) but each was trained on gait data at varying speeds. The respective training and testing speed ranges for each of the models were as follows: (1) the generalised-speed model was trained and tested on all gait speed ranges, (2) the high-speed model was trained on medium and high speeds and tested on low speeds, (3) the low-speed model was trained on low and medium speeds and tested on high speeds, and (4) the low-high-speed model was trained on low and high speeds and tested on medium speeds. Details about the FCNN models and their corresponding training and testing speed ranges can be found in Table 1.

**Figure 2:**
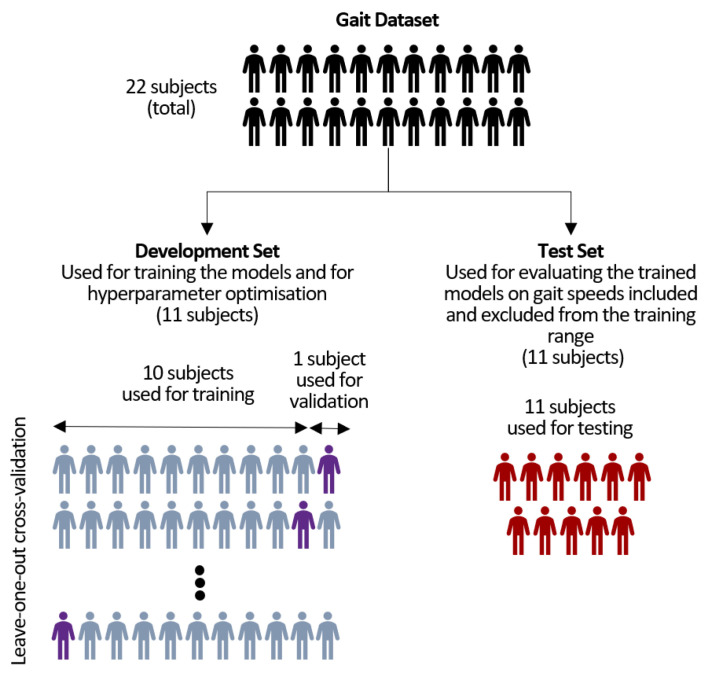
Illustration of the train-test split used for training and evaluating the FCNNs using the leave-one-out cross-validation method.

The data from the 22 able-bodied individuals were randomly split into two sets: a development set and a test set. This division was performed at the subject level, with 11 subjects in each set. The development set was used for two main purposes: hyperparameter optimisation and model training. For hyperparameter optimisation, 70% of the subjects in the development set were used for training (8 subjects), whereas the remaining 30% (3 subjects) were used for validation. After the hyperparameter optimisation phase, cross-validation was performed using the leave-one-subject-out method. In this process, the FCNNs were trained on data from 10 subjects in the development set, with the data from the 11th subject left out for validation. This procedure was repeated 11 times, each time using a different subject as the validation subject. The test set, consisting of data from unseen subjects, was used to evaluate the performance of the models each time (see Figure 2). This evaluation included assessing the performance of the models on the gait speeds included in the training range, as well as on outlier speeds.

The windowing method was used to generate training and testing samples for each set in the study [32]. The stride length, which determines the number of training samples that will be derived from each gait sequence, was varied for the different FCNN models to ensure that all models, including the generalised-, low-, high-, and low-high-speed models, were trained on a similar number of samples. Each training sample consisted of an input matrix, $x_{in}$, and target vector $y_{out}$. $x_{in}$ represents a 200-time-step window of joint angle values, including the hip, knee, and ankle angles for the left and right legs in the sagittal plane. The input window corresponds to 1 s of data for a sampling frequency of 200 Hz. $y_{out}$ represents the values of the joint angles (hip, knee, and ankle angles for the left and right feet) for the next time-step. The output window size is 1 time-step, representing the immediate future joint angle values that the FCNN models are trained to predict.

For *n* samples in a set, $X_{in}\in R^{n\times l_{in}\times f}$, $l_{in}$ (set to 200) is the input window size and *f* (set to 6) is the number of input features (kinematic joint angles). Similarly, in $Y_{out}\in R^{n\times l_{out}\times f}$, $l_{out}$ (set to 1) is the output window size and *f* (set to 6) is the number of output features.

The FCNN models (generalised-speed, low-speed, high-speed, and low-high-speed models) were trained and tested on different gait speeds. All inputs to the models and the corresponding target outputs were normalised using min-max normalisation such that $X_{in}\in \left[0,1\right]$ and $Y_{out}\in \left[0,1\right]$. The min-max values were chosen to accommodate for the minimum and maximum values of all the individuals in the dataset, with an additional safety boundary to ensure the data fell within the normalised range.

### 3.4. Model Architecture and Optimisation

**Figure 3:**
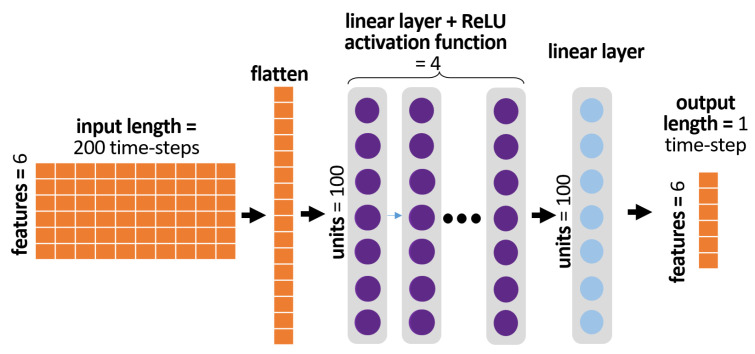
Fully connected neural network (FCNN) architecture.

In a previous study of ours, the fully connected neural network (FCNN) demonstrated low errors in both short-term and long-term gait trajectory prediction tasks and exhibited higher robustness to added noise [33]. These were the reasons for selecting the FCNN for this study. The input to the FCNN, which was 2-dimensional $R^{200\times 6}$ as it included the values of 6 joint angles for a 200-time-step window, was flattened into a 1-dimensional vector $R^{1200}$ and passed through 5 fully connected linear layers with ReLU activation functions in between. The FCNN architecture is illustrated in Figure 3.

For training, each of the models was optimised to minimise the mean squared error (MSE) difference between the predictions and the target one-step-ahead kinematic values using the Adam optimiser. The optimal hyperparameters, including the learning rate, number of layers, number of nodes per layer, and batch size, were selected based on the tree-structured Parzen estimator algorithm, a type of Bayesian hyperparameter sampler. Hyperparameter optimisation (HPO) was applied to the generalised model, and the optimised architecture resulting from this process was used for the other models (low-speed, high-speed, and low-high-speed models). The search space and corresponding selected values are shown in Table 2.

During training, the dynamic time-warping (DTW) distances were calculated between 200 recursively predicted time-steps and the true gait values after each training epoch. The DTW distances were used as a metric to determine when to end the training of the models. The models were trained for 70 epochs, with training being terminated earlier (using the early stopping method) if the DTW distances on the validation set did not decrease for 20 epochs. In our previous study [33], we elaborated on how DTW distances are used to optimise gait trajectory prediction models.

The Pytorch machine learning framework was used in this study, along with various libraries, including Matplotlib, Numpy, Seaborn, SciPy, Scikit-Posthocs, and Optuna, for hyperparameter optimisation [34]. The DTW Python package was used for calculating dynamic time-warping distances [35]. The computations were performed using an Nvidia Geforce RTX 2070 GPU.

### 3.5. Evaluation Metrics and Statistical Analysis

The mean absolute error (MAE) and mean squared error (MSE) were used as the evaluation metrics to assess the performance of the models. These metrics were calculated for both short-term predictions, comparing one predicted time-step to the actual gait values, and long-term predictions, comparing 200 time-steps of gait values generated using recursive forecasting (where predictions are fed back as input to generate further predictions) to the actual gait values. The MAEs and MSEs were calculated after the de-normalisation of the models’ outputs. The MAE and MSE formulas for *f* features, *n* test samples, and $l_{out}$ output prediction lengths (set to 1 for short-term predictions and to 200 for long-term predictions) are:

Mean absolute error (MAE):

(1) $$MAE=\frac{1}{nfl_{out}}\sum_{i=1}^{n}\sum_{j=1}^{f}\sum_{k=1}^{l_{out}}\left|y_{i,j,k}-{\hat{y}}_{i,j,k}\right|$$

Mean squared error (MSE):

(2) $$MSE=\frac{1}{nfl_{out}}\sum_{i=1}^{n}\sum_{j=1}^{f}\sum_{k=1}^{l_{out}}{\left(y_{i,j,k}-{\hat{y}}_{i,j,k}\right)}^{2}$$

In Section 4, we report the results of the statistical tests we conducted. The tests were carried out in Python using the Scipy and Scikit-Posthocs libraries.

## 4. Results

**Figure 4:**
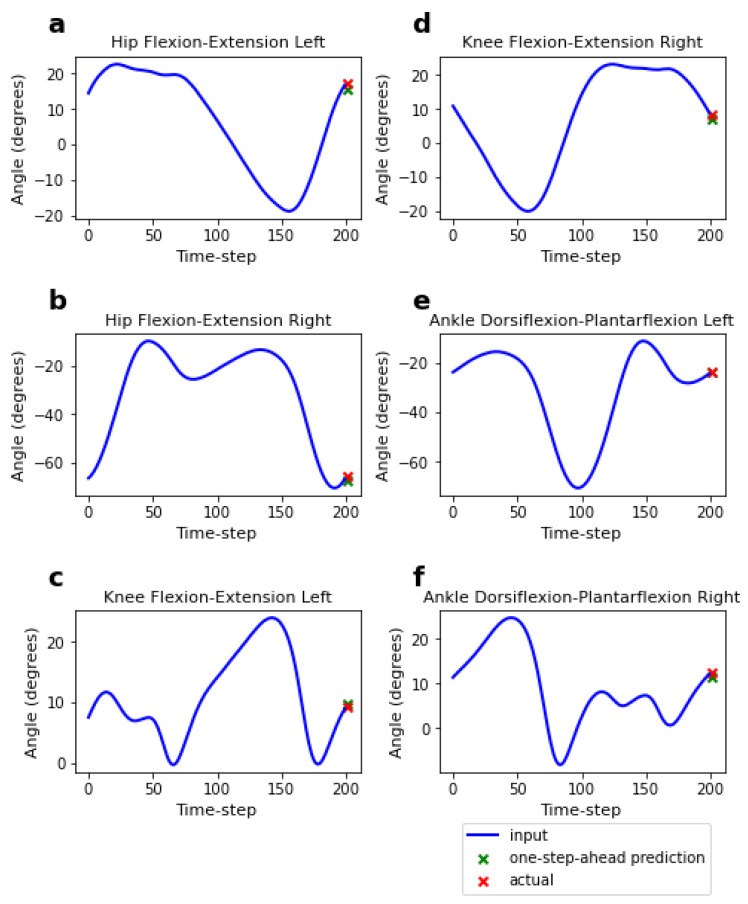
Short-term (one-step-ahead) prediction of the flexion-extension angles of the hip, knee, and ankle. Predictions (green marks) are made based on a 200-time-step input to the model (blue lines) and then compared to the actual values (red marks).

**Figure 5:**
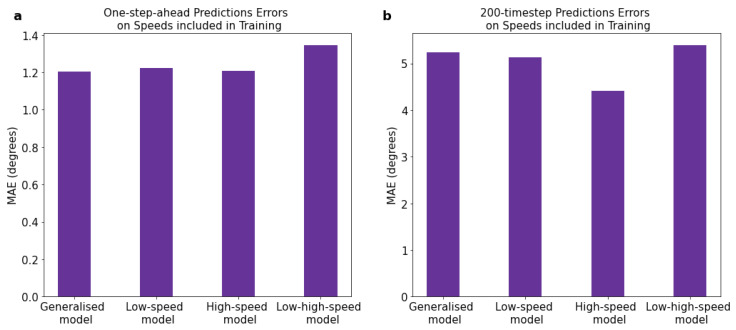
Prediction errors on speeds included in training range. (**a**) Errors in short-term (1-step-ahead) predictions. (**b**) Errors in long-term (200-time-step) recursive predictions.

**Figure 6:**
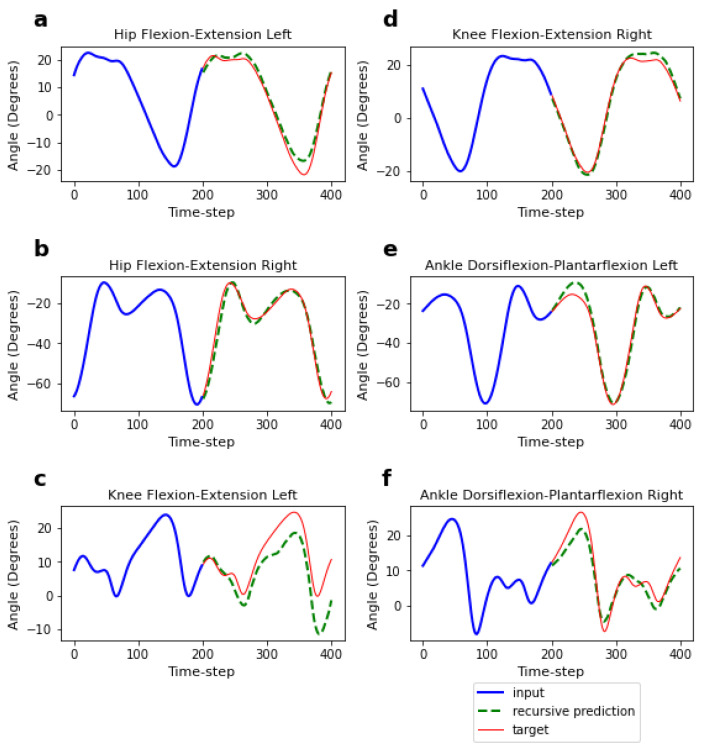
Long-term (200-time-step) prediction of the flexion-extension angles of the hip, knee, and ankle. Recursive predictions (green lines) are made based on a 200-time-step input to the model (blue lines) and then compared to the actual values (red lines).

We first evaluated the performance of the four different models (generalised-speed, low-speed, high-speed, and low-high-speed models) on speeds included in the training range but from unseen subjects. The MAEs and MSEs for the short-term (1-time-step) and long-term (200-time-step) predictions are presented in Table 3. For the short-term predictions (depicted in Figure 4), the MAEs ranged from 1.21 to 1.34°, with a maximum MAE difference of 0.14° across the four models. The low-speed model showed slightly higher errors compared to the other three models (see Figure 5a, illustrating the MAE difference for short-term predictions). Meanwhile, for the long-term predictions (depicted in Figure 6), the MAEs were higher, ranging from 4.42 to 5.39°, with a maximum difference of 0.97° across the four models. The low-high-speed model exhibited the highest MAEs, whereas the high-speed model exhibited the lowest MAEs (see Figure 5b, illustrating the MAE difference for long-term predictions). All differences in the one-step-ahead prediction errors on speeds included in the training range among the various FCNNs were statistically significant. The statistical significance was determined based on the Kruskal–Wallis H-test (*p* < 0.05), followed by Dunn’s post hoc test for pairwise comparisons.

**Figure 7:**
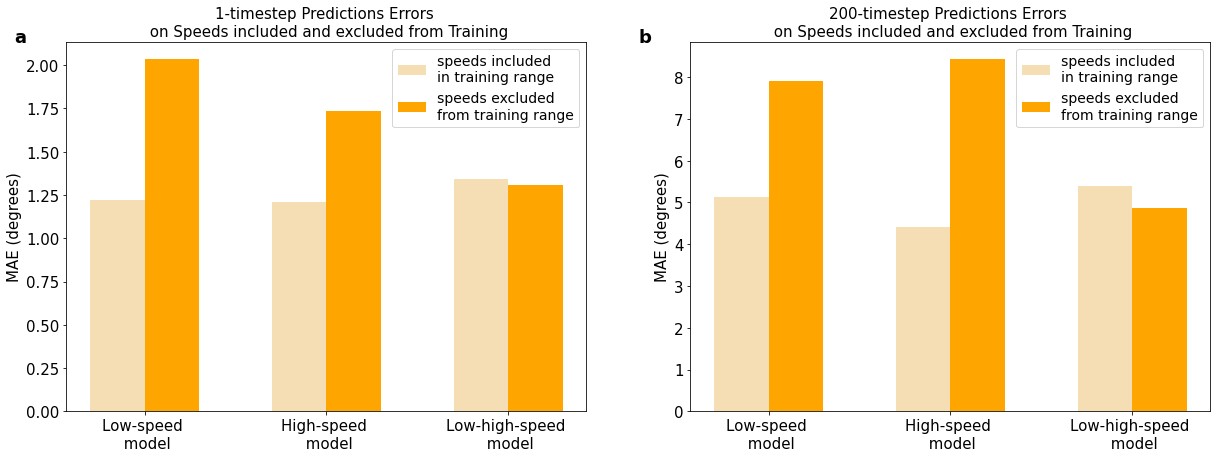
Comparison of prediction errors between gait speeds included in the training range and excluded speeds. (**a**) Errors in short-term (1-step-ahead) predictions. (**b**) Errors in long-term (200-time-step) recursive predictions. All differences in prediction errors (MAEs) between included and excluded speeds for each FCNN model are statistically significant for both short-term and long-term predictions (significance determined using the Kruskal–Wallis H-test (*p* < 0.05)).

We then evaluated the performance of the four models on speeds excluded from the training range and from unseen subjects. The MAEs and MSEs for the short- and long-term predictions on excluded speeds are presented in Table 4. For the short-term predictions on excluded speeds, the MAEs ranged from 1.31 to 2.03° across the four models, and from 4.86 to 8.42° for the long-term predictions. We compared the difference in the performance of the models on speeds included in the training range and speeds excluded from the training range (see Figure 7). It was observed that the performance of the low- and high-speed models worsened when tested on the excluded speeds. For the one-step-ahead predictions, the MAE of the low-speed model on the excluded speeds was 66.2% higher compared to the MAE on speeds included in the training range, whereas the MAE of the high-speed model was 43.7% higher. For the long-term predictions, the MAE of the low-speed model on the excluded speeds increased by 54.3% compared to the MAE on the included speeds, whereas the MAE of the high-speed model increased by 90.7%. However, the low-high-speed model showed different outcomes. It performed better on the excluded speeds (i.e., medium-speed ranges) compared to the included speeds (i.e., low- and high-speed ranges). In fact, the MAE for the excluded speeds decreased by 2.8% for the short-term predictions, compared to the MAE for the included speeds, and by 9.8% for the long-term predictions. All differences in the prediction errors (MAEs) between the included and excluded speeds for each FCNN model were statistically significant for both the short- and long-term predictions. Statistical significance was determined based on the Kruskal–Wallis H-test (*p* < 0.05).

## 5. Discussion

In this study, fully-connected-neural networks (FCNN) were implemented for gait trajectory prediction. The FCNNs were trained on four different gait speed ranges and evaluated on speeds included in and excluded from the training set. The results showed that the performance of the generalised-, low-, high-, and low-high-speed models was very similar when evaluated on the speeds they were trained on. The low-high-speed model exhibited slightly higher errors compared to the other three models for both short-term and long-term predictions. On the other hand, the high-speed model exhibited slightly lower errors, especially for long-term predictions. The findings are consistent with the results of a previous study by Zaroug et al. [21], which reported improved performance for lower limb kinematic predictions at higher gait speeds.

In comparison to related studies, Kang et al. [13] reported good performance of their gait-phase estimation model on extrapolated speeds, although they only evaluated speeds slightly higher than their training speed ranges. Meanwhile, Lu et al. [28] evaluated their continuous gait-phase recognition algorithm on untrained speeds and observed a decline in performance, but their results were based on a small number of subjects. In this study, the performance of the low- and high-speed FCNN models worsened when evaluated on excluded speeds, as indicated by an increase in mean squared errors (MSEs) and mean absolute errors (MAEs) for both short-term and long-term predictions. The MAEs increased by 43.7% to 90.7% for the low- and high-speed models when compared to the MAEs on trained speeds. Interestingly, the low-high-speed model, which was trained on low- and high-speed ranges only, performed well on medium speeds. In fact, the MAEs for medium speeds improved by 2.8% for short-term predictions and 9.8% for long-term predictions compared to the low- and high-speed ranges the model was trained on. These results suggest that FCNNs are capable of interpolating to speeds that lie between the maximum and minimum training speeds, even if they have not been explicitly trained on those speeds. However, they are unable to extrapolate to speeds beyond or below the maximum and minimum speeds of the training range.

One important limitation of our study is the relatively small size of the dataset used to develop and test our models, which included data from 22 subjects. This limitation is a common challenge for many applications involving human data, as data scarcity often arises due to practical constraints and limited resources. To mitigate the risk of overfitting our models and assess their generalisability across different subjects, we implemented leave-one-out cross-validation. Nonetheless, we still need to validate our findings with FCNNs that are trained on larger datasets. Advancements in wearable technology that enable continuous data collection may assist in addressing the issue of data scarcity. Another limitation of our study is that the data used to train the models were collected from gait cycles performed at constant speeds. To improve the implementation, it would be beneficial to include data from gait cycles with dynamic speeds, as this better reflects real-life walking conditions. Additionally, the study only considered data captured on even surfaces, without accounting for inclinations or unevenness that may be encountered in outdoor environments. Furthermore, the gait data used in the study were captured using a motion capture system. In practical applications such as exoskeletons, data would typically be obtained from onboard wearable sensors such as inertial measurement units (IMUs) or built-in encoders.

## 6. Conclusions

This study explored the performance of fully connected neural networks (FCNNs) in predicting gait trajectories across different speed ranges. It examined both short-term and long-term predictions and evaluated the models on speeds that were both included and excluded from the training gait speed range. The results revealed that the FCNN models exhibited a decline in performance when predicting kinematic joint trajectories at gait speeds significantly higher or lower than the training speed ranges. However, the FCNN models demonstrated satisfactory performance on speeds within the maximum and minimum speed ranges, even if those speeds were not included in the training dataset. These findings highlight the importance of considering the range of speeds that an exoskeleton may encounter in real-life applications during the training and development of the models. They also emphasise the need for the development of explainable AI techniques to gain insights into the influential input features and limitations affecting the model’s performance. This information can enrich our knowledge of gait analysis and biomechanics, leading to improved interventions for gait assistance and rehabilitation. 

## Figures and Tables

**Table 1:** Range of speeds FCNNs were trained and tested on (low speeds 0.5–1.0 m/s, medium speeds 1.05–1.45 m/s, and high speeds 1.5–1.85 m/s).

Model	Training Set Speed Range (m/s) (Included Speeds)	Testing Set Speed Range (m/s) (Excluded Speeds)
Generalised-speed model	all (0.5–1.85)	all (0.5–1.85)
Low-speed model	low and medium (0.5–1.45)	high (1.50–1.85)
High-speed model	high and medium (1.05–1.85)	low (0.5–1.0)
Low-high-speed model	low and high (0.5–1.0, 1.50–1.85)	medium (1.05–1.45)

**Table 2:** Search space for FCNN hyperparameters and the selected values.

Hyperparameter	Search Space	Selected Value
learning rate	[0.01, 0.001, 0.0001, 0.00001]	0.00001
number of layers	[3, 4, 5, 8, 10, 12]	5
nodes per layer	[10, 30, 70, 100, 150, 200]	100
batch size	[32, 64, 128, 256, 512]	32

**Table 3:** MSEs and MAEs for 1-step-ahead and 200-time-step gait trajectory predictions for generalised-, low-, high-, and low-high-speed models evaluated on speeds included in the training range (in degrees).

		Generalised-Speed Model	Low-Speed Model	High-Speed Model	Low-High-Speed Model
1 time-step	MSE	2.58	2.66	2.62	3.43
	MSE std	0.99	1.08	1.32	2.58
	MAE	1.21	1.22	1.21	1.34
	MAE std	0.24	0.25	0.30	0.46
200 time-steps	MSE	60.84	58.36	43.13	63.25
	MSE std	25.97	22.00	24.18	23.92
	MAE	5.24	5.13	4.42	5.39
	MAE std	1.04	0.98	1.06	0.97

**Table 4:** MSEs and MAEs for 1-step-ahead and 200-time-step gait trajectory predictions for low-, high-, and low-high-speed models on speeds excluded from the training range (in degrees).

		Low-Speed Model	High-Speed Model	Low-High-Speed Model
1 time-step	MSE	7.40	5.41	3.21
	MSE std	4.64	3.18	2.50
	MAE	2.03	1.74	1.31
	MAE std	0.63	0.54	0.44
200 time-steps	MSE	142.49	152.09	49.85
	MSE std	107.71	65.94	21.87
	MAE	7.92	8.42	4.86
	MAE std	3.06	2.02	1.06

## Data Availability

The dataset used in this study has been collected by Camargo et al. [29] and publically shared at https://www.epic.gatech.edu/opensource-biomechanics-camargo-et-al (accessed on 1 December 2022).

## Abbreviations

The following abbreviations are used in this manuscript:

AI	Artificial Intelligence
DTW	dynamic time warping
FCNN	fully connected neural network
MAE	mean absolute error
MSE	mean squared error
